# Supplementary material for: A comprehensive genome-wide cross-trait analysis of sexual factors and uterine leiomyoma
Source: PLoS Genet. 2024 May 3;20(5):e1011268. doi: 10.1371/journal.pgen.1011268 (PMC11095738; doi:10.1371/journal.pgen.1011268)
Supplement: S1 Text — (DOCX) [file pgen.1011268.s001.docx]

**Supplementary Methods for mediation analysis:**

To investigate the potential mediating role of hormone-related phenotypes, including age at first birth (AFB), age at menarche (AAM), age at natural menopause (ANM), sex hormone-binding globulin (SHBG), SHBG adjusted for body mass index (SHBGadj), total testosterone (TT), bioavailable testosterone (BT), and estradiol (E2), in the relationship between sexual factors and uterine leiomyoma (UL), we conducted a mediation analysis within a two-step Mendelian randomization (MR) framework (**S1 Figure**).

We obtained the largest available genome-wide association study (GWAS) summary statistics for each mediator under investigation (details on data sources can be found in **S4 Table**). We employed univariable MR to estimate the effect of the exposure trait (AFS/NSP) on the outcome trait (UL risk), referred to as the total effect C, as well as the effect of the exposure trait on each potential mediator, termed the effect A. We applied multivariable MR to assess the effect of each potential mediator on the outcome trait (effect B), while accounting for additional adjustments for the exposure. The proportion mediated (PM), representing the total effect of the exposure on the outcome that is mediated by the mediator, was calculated by dividing the indirect effect (the multiplication of effect A and effect B) by the total effect C. Standard errors for PM were estimated using the delta method. As in our main MR analysis, statistical significance was determined using a Bonferroni-corrected P-value threshold of 0.025 to account for the two sexual factors tested.

**Interpretation and discussion of the mediation analysis results:**

We emphasize that two issues need to be taken into consideration while interpreting our mediation results. First, in the AFS-AFB-UL analysis, we observed an indirect effect that is larger than the total effect (**S9 Table**). This is likely due to a very small (close to zero) population direct effect of AFS on UL. In our sample, the null direct effect came out positive due to sample variability, resulting in an indirect effect stronger than the total effect (the sum of indirect and direct effects in opposite directions). This interpretation aligns with our univariable MR analysis, which found no evidence supporting a causal effect of AFS on UL risk.

Second, in analyses involving AFS-AFB/AAM/ANM-UL, we observed significant indirect effects in the absence of a significant total effect (**S9 Table**). In a mediation model, the standard error of the indirect effect depends on the sample variability of Effect A and Effect B, while the standard error of the total effect depends on the variability of both the indirect effect (including Effect A and Effect B) and the direct effect. With more components involved, there is more uncertainty, making it less likely to find the total effect as significant compared to finding the indirect effect significant. Thus, one possible interpretation for our findings is that we lack sufficient evidence to demonstrate that AFS influences UL, but if an influence is detected, it is likely due to the intervening effect of AFB (other factors such as AAM and ANM may also contribute, albeit to a lesser extent), rather than a direct causal relationship between AFS and UL risk. This interpretation aligns with our multivariable MR analysis, where the observed significant effect of AFS on UL in the multivariable models became non-significant when AFB was additionally included.

Ideally, measuring sex hormones during the period when individuals typically experience their first sexual intercourse would provide a more direct assessment of the potential mediating effect of sex hormones in the AFS-UL relationship. However, in the case of UK Biobank participants, the median AFS is 18, while the median age at recruitment (when sex hormone levels were measured) is 57. We acknowledge this limitation and recognize that our inability to perform participant-level analysis using sex hormone levels measured among younger populations, or summary-level analysis using age-stratified GWAS data for sex hormone traits, is due to data constraints. Future studies leveraging data from participants within the specific age group in question are necessary to refine our understanding of how sexual factors, sex hormones, and UL risk interact during different life periods.
