# Supplementary material for: A comprehensive genome-wide cross-trait analysis of sexual factors and uterine leiomyoma
Source: PLoS Genet. 2024 May 3;20(5):e1011268. doi: 10.1371/journal.pgen.1011268 (PMC11095738; doi:10.1371/journal.pgen.1011268)
Supplement: S1 Fig — (DOCX) [file pgen.1011268.s002.docx]

**S1 Figure.** Graphical representation of proposed mediation through hormone-related phenotypes in the association of sexual factors with uterine leiomyoma.
