# Supplementary material for: A comprehensive genome-wide cross-trait analysis of sexual factors and uterine leiomyoma
Source: PLoS Genet. 2024 May 3;20(5):e1011268. doi: 10.1371/journal.pgen.1011268 (PMC11095738; doi:10.1371/journal.pgen.1011268)

**S2 Figure.** Clustering of cell-type-specific annotation for uterine leiomyoma, age at first sexual intercourse, and lifetime number of sexual partners over histone marks. Each colored square reflects the z-score, scaled by traits. Red indicates enrichment, blue indicates depletion. Deeper color represents stronger magnitude of effects. Asterisks represent statistical significance withstanding correction (*P* < 0.05/396). (A). DNase, (B). H3K27ac, (C). H3K36me3, (D). H3K4me1, (E). H3K4me3, and (F). H3K9ac.

UL, uterine leiomyoma; AFS, age at first sexual intercourse; NSP, lifetime number of sexual partners.


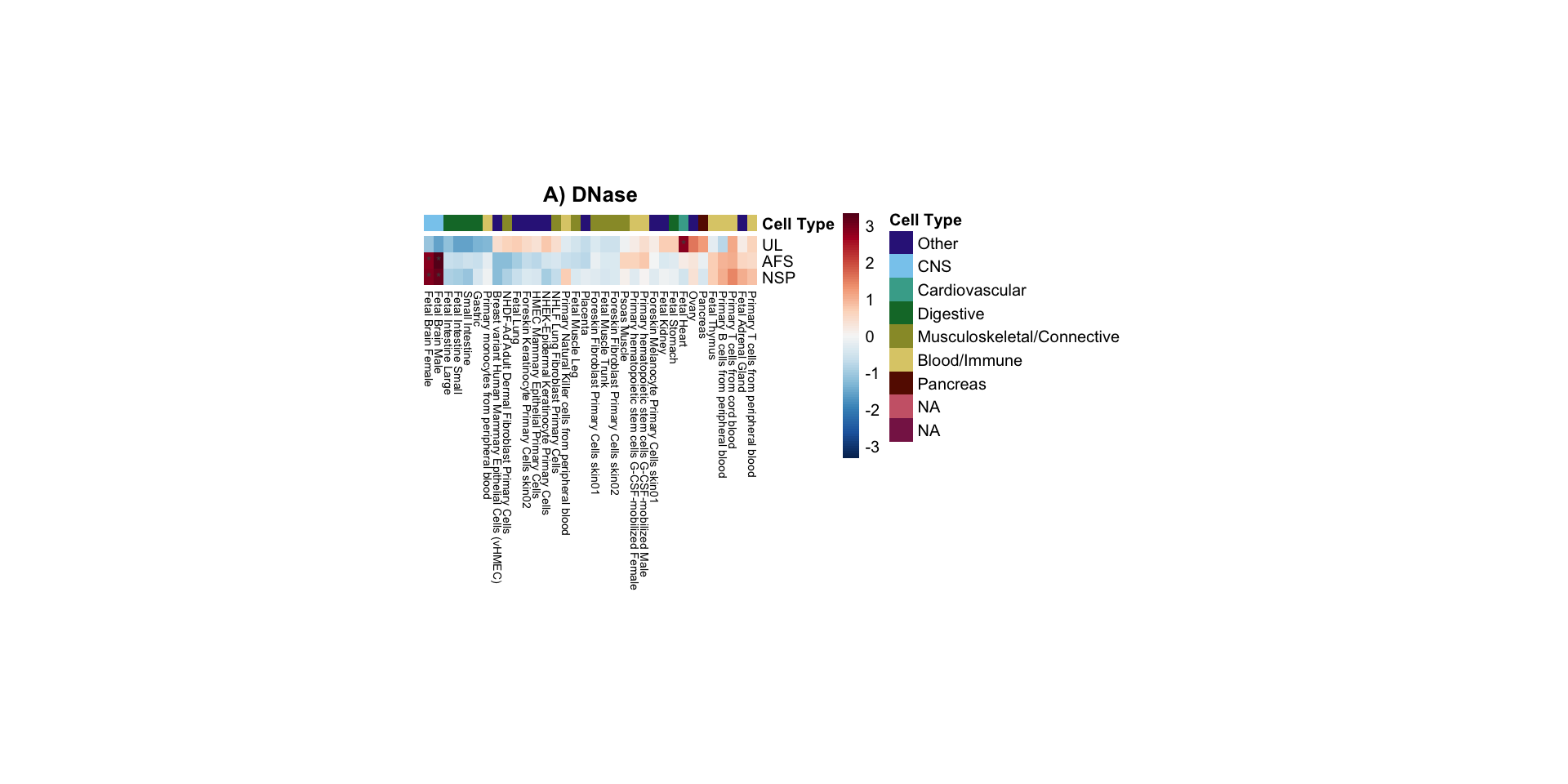


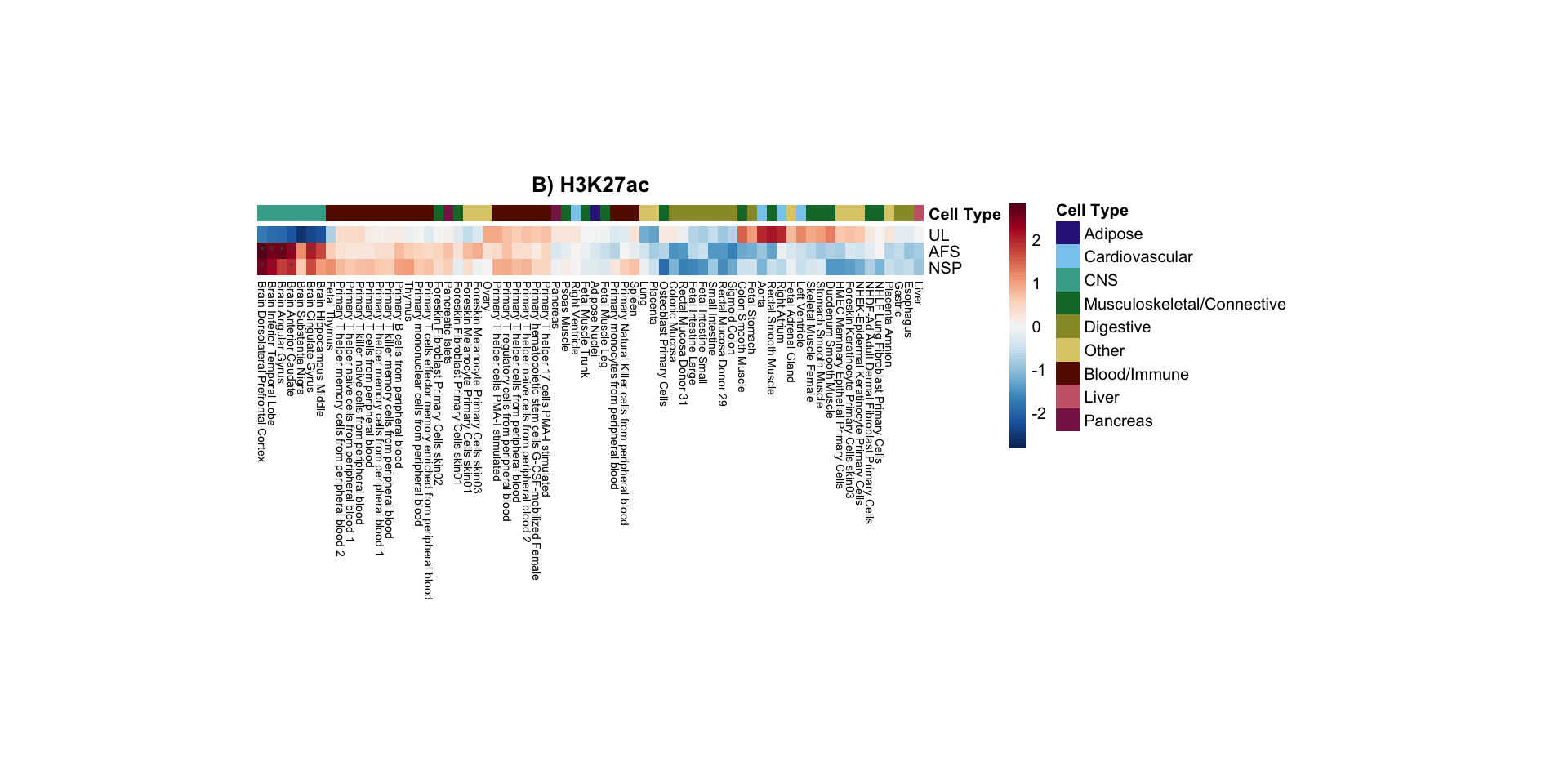


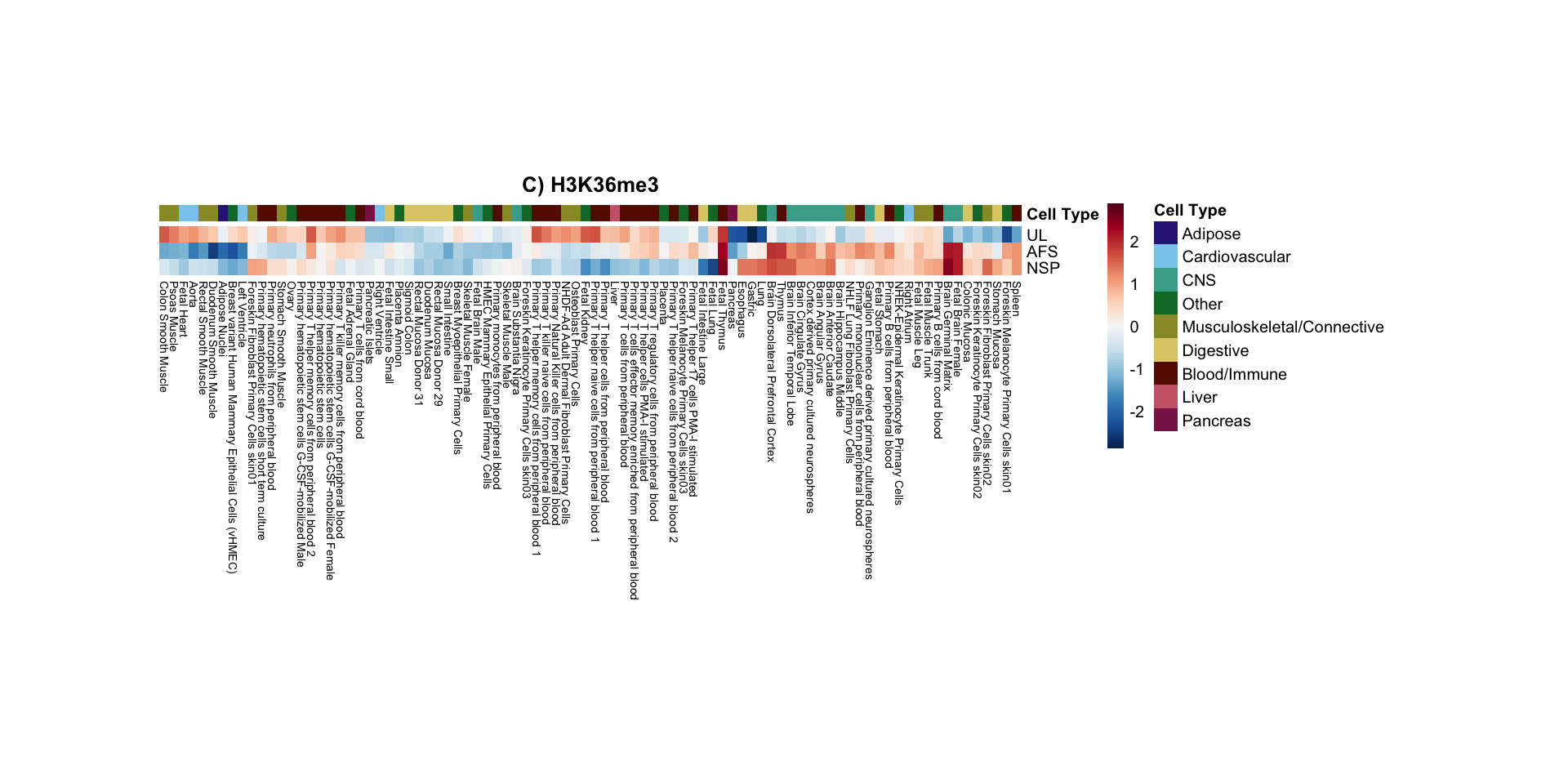


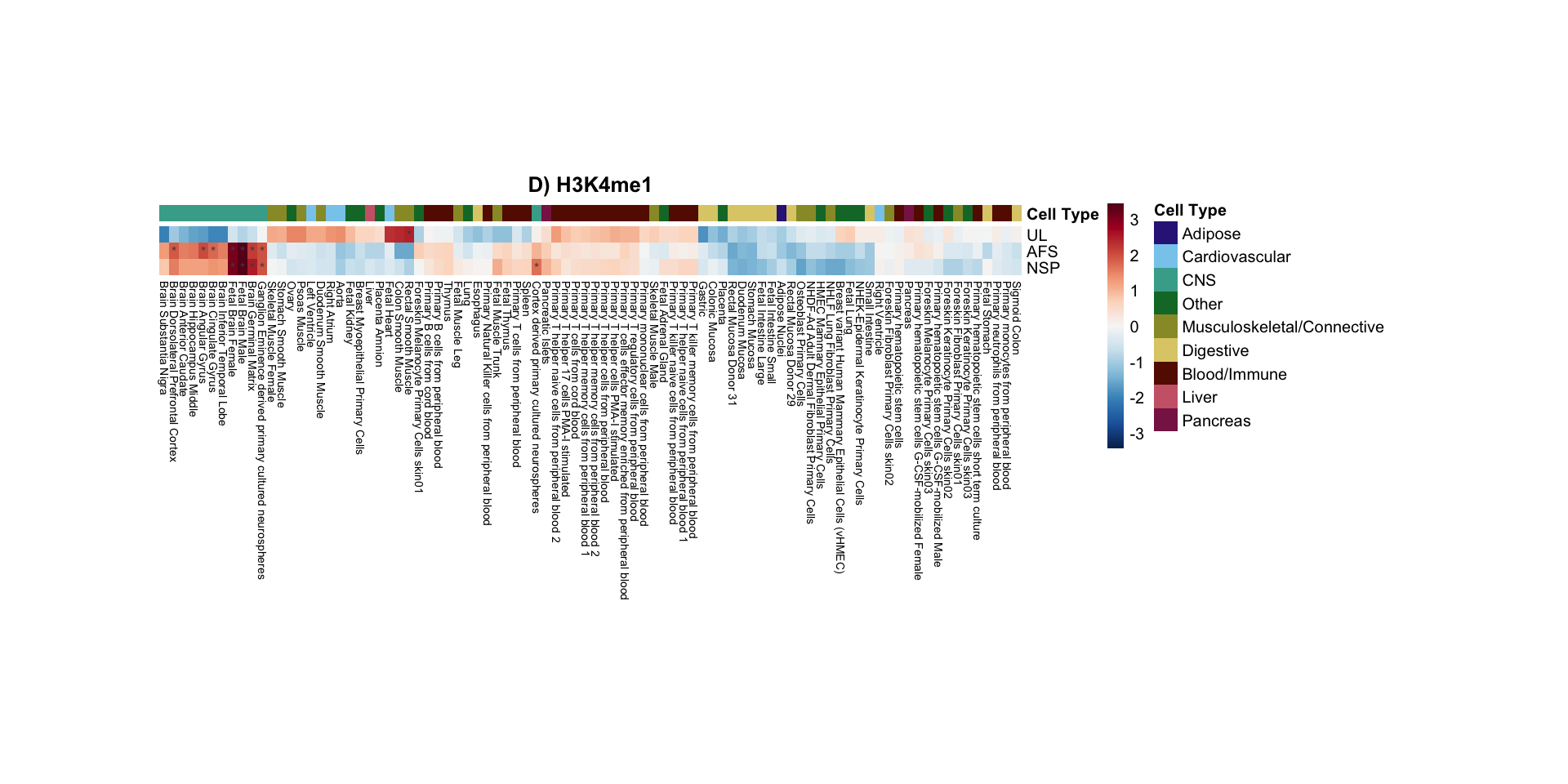


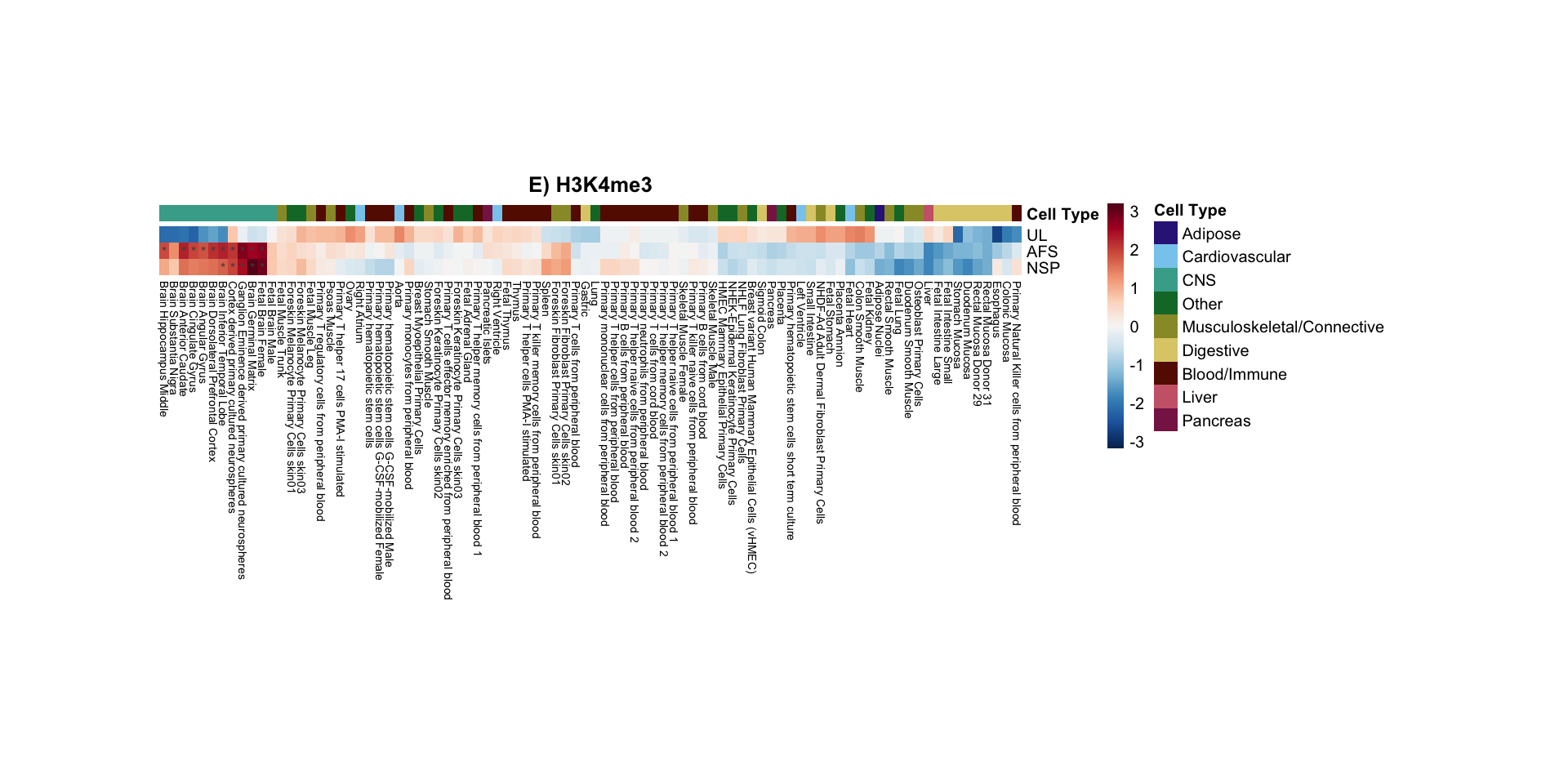


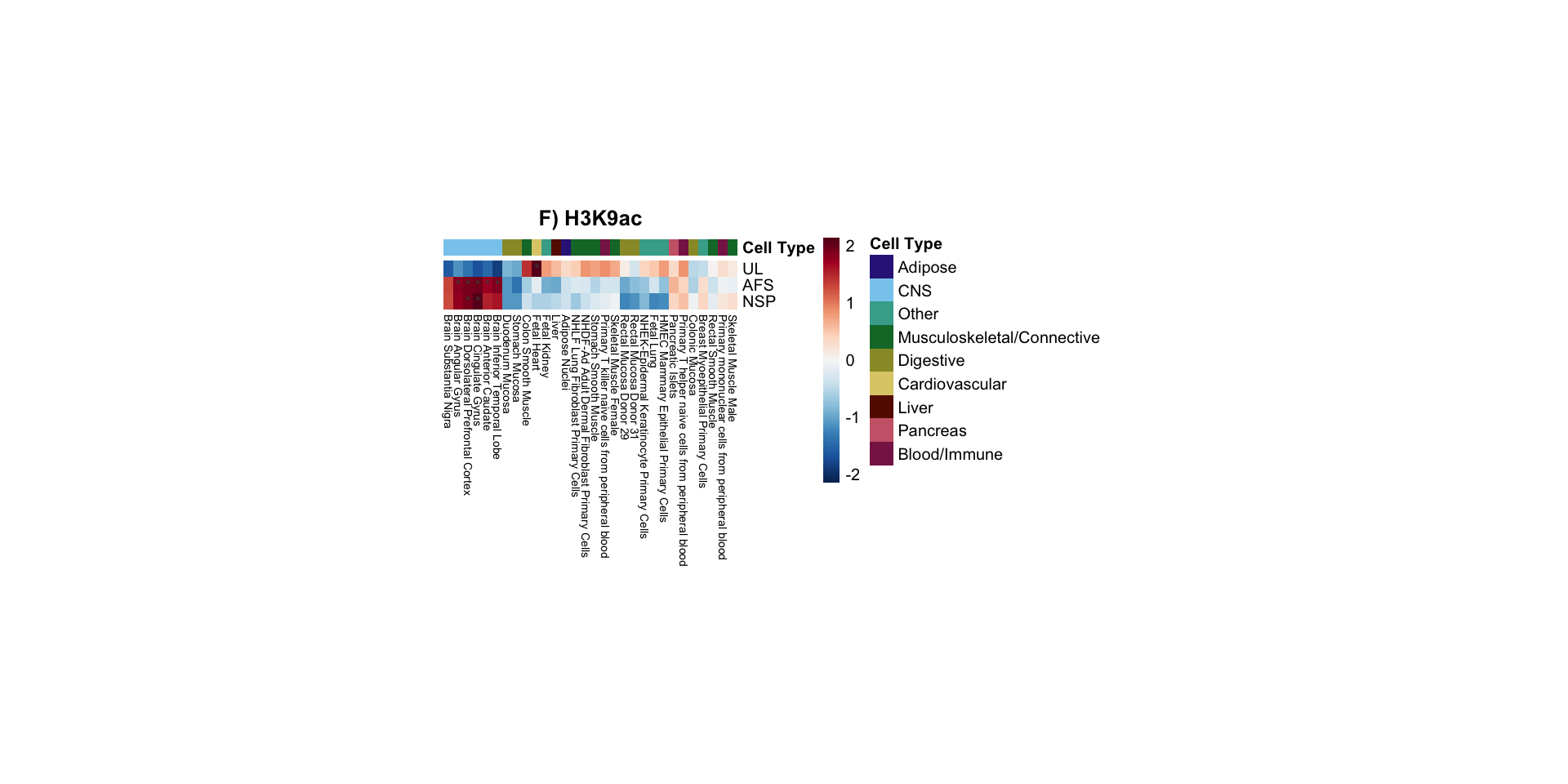

Supplement: S2 Fig — (DOCX) [file pgen.1011268.s003.docx]
