# Supplementary material for: A comprehensive genome-wide cross-trait analysis of sexual factors and uterine leiomyoma
Source: PLoS Genet. 2024 May 3;20(5):e1011268. doi: 10.1371/journal.pgen.1011268 (PMC11095738; doi:10.1371/journal.pgen.1011268)
Supplement: Attachment — Submitted filename: PGENETICS-D-23-01080.docx [file pgen.1011268.s013.docx]

*A comprehensive genome-wide cross-trait analysis of sexual factors and uterine leiomyoma*

Review of PGENETICS-D-23-01080

**Summary**

Uterine leiomyomas are a common morbidity in women. They are known to be associated with multiple female reproductive characteristics, including parity and age at menarche. However, the impact of more environment (behavior) reproductive factors, including the characteristics focused on in this paper (age at first sexual intercourse and lifetime number of sexual partners), is unclear. The authors hypotheses that age at first sexual intercourse and the lifetime number of sexual partners could since they could increase individuals’ exposures to infectious agents. They used publicly available genome-wide summary statistics from large European ancestry specific studies of age at first sexual intercourse, lifetime number of sexual partners, and uterine leiomyomas. They performed numerous statistical analyses, including global and local genetic correlation analyses, cell-type-specific enrichment analysis, cross-trait meta-analysis, and one- and two-sample Mendelian randomization. They conclude that their findings provide evidence of considerable overlapping biology, highlighted genetic correlations, clustering of cell-type-specific annotations, and the presence of shared loci across traits. However, they did not find any evidence to support a causal relationship between age at first sexual intercourse and uterine leiomyomas or number of lifetime sexual partners and uterine leiomyomas. The numerous analyses and approach are unique. However, it is not of broad interest to researchers in genetics and genomics and lacks truly rigorous methodology. I finding this manuscript lacking and concerning for multiple reasons which I highlight below.

**Major comments and questions**

- Though I understand the authors’ logic for taking a genetic approach to this research question (e.g., observational studies are lacking, susceptible to bias and reverse causality, etc.), there are serious concerns about the approach. There are innumerable issues with using genetic instruments to study characteristics (age at first sexual intercourse and lifetime number of sexual partners) that are known to be heavily influenced by the environment. Though the authors discuss the issue of pleiotropy, they do not provide discussion of the enormous effects of the environment (behavior, culture, religion, etc.) on both the sexual factors and uterine leiomyomas.
- Both the two-sample and one-sample Mendelian randomization demonstrated no causal effects. Yet, the authors heavily highlight the non-causal association analyses. Framing of the results and discussion as common biological mechanisms is misleading as the study found correlation, not causation.
- Ignoring the issues in using genetics for age at first sexual intercourse and number of lifetime sexual partners, I am not convinced that this study would lead to any significant changes in individuals’ decision-making or clinical practice. There is also concern about stigmatization.
- Also, the SNP heritability of the three highlighted variables, particularly AFS and NSP, is incredibly low. The authors state that the SNP heritabilities (13%, 12-23%, 13%) suggest a non-trivial genetic background for each trait. I would argue that it suggests an even larger role of environmental factors. The second paragraph of the introduction does not provide adequate support for the use of genetics and genetic epidemiologic methods to study these relationships. It undermines it.
- The authors’ argument for the connection between uterine leiomyomas, age at first sexual intercourse, and number of lifetime sexual partners seems to be heavily focused on infection. It is unclear why the authors didn’t instead focus on infection instead of these apparent proxies for exposure to infection. The approach the authors take suggests they are equating earlier age at first sexual intercourse and higher number of lifetime sexual partners to higher risk/more likely to have uterine infections. This is a broad generalization (an individual could have both things but take precautions/use protection and it is heavily dependent on individuals’ partner.
- The study only included summary statistics from genome-wide association studies conducted in European populations, greatly diminishing the generalizability of the study results.
- The authors utilized publicly available genome-wide summary statistics for NSP that contained females and males, though the outcome they are interested in (uterine leiomyoma) can only occur in those biologically female with uteruses. With no mention of the proportion of the cohort that was male, it is impossible to conclude if their results are biased due to heterogeneity by sex. Furthermore, with the uterine leiomyoma genome-wide association study, there is no discussion of whether females who had hysterectomies were excluded or the percent of the cohort whose phenotype was ascertained by self-report versus clinical documentation (which could introduce bias).
- The genome-wide association studies for sexual factors both used poorly worded questions to gauge the outcomes, as they did not clarify which type of sexual intercourse they meant or what participants would consider sexual intercourse. The ascertainment methods are particularly crucial for the reviewed study as certain types of sexual contact would likely not increase (or not to the same amount) risk of uterine infections, which the authors appear to argue is the most likely mechanism through which these factors causally impact uterine leiomyomas.
- There are concerns with the Mendelian randomization analyses. The authors performed bi-directional two-sample Mendelian randomization, evaluating the potential for reverse causation. However, this approach for age at first sexual intercourse is flawed. Age at first sexual intercourse likely occurs early in life (e.g., teens or twenties) for most individuals, whereas, development or diagnosis of uterine fibroid, though the vast majority of females have them by age 50, likely occurs later. The percent of females diagnosed before their first sexual intercourse is undoubtedly very small. This causal analysis is invalid due to this incorrect time/order relationship.
- In some areas of the paper, the authors use ancestry terminology while other areas they use sociocultural definitions of race and ethnicity (e.g., European ancestry on page 5, white descent on page 8). Their choice in terminology for sex is also flawed, switching between biological sex and gender (e.g., females versus women). Please consider changing the language throughout the paper to the correct terminology.
- Mediation analyses would further clarify that the relationships are not being driven by actual biologically controlled factors.
- A follow-up analysis using a study that meticulously defined their cohorts and uterine leiomyomas, such as the genome-wide association studies by Edwards et al or Hellwege et al which contained only cases and controls that had pelvic imaging, would increase the rigor of this study. As is, the study could be biased due to ascertainment of uterine leiomyomas. Since they provide no detail on the proportion whose case status was ascertained via health records versus self-report, the possibility of the study suffering from recall bias cannot be determined.

**Minor comments and questions**

- The abstract does not include sufficient information summarizing why the authors hypothesize that age at first sexual intercourse and lifetime number of sexual partners influence uterine leiomyomas. The first sentence of the author summary provides a clear description of the potential mechanisms and should be included in the abstract.
- Important details of the genome-wide association studies that were utilized were not provided or are provided only in supplementary material. More details of the genome-wide association analyses should appear in the manuscript itself, such as what each study adjusted for (principal components, race/ethnicity, age at measurement, etc.).
- More discussion on how the genome-wide association studies defined their phenotype and adjusted their analyses is necessary.
- In the cross-trait meta-analysis section of the methods, the “Significant pleiotropic SNPs” and “Novel pleiotropic SNPs” do not need to be in bold. The rsIDs in the results also do not need to be bolded.
- The authors state they were able to perform separate analyses for pre- and post-menopausal women but did not state how menopause was defined or ascertained. Additional details of these analyses would be useful.
- Please consider combining some paragraphs, as some are composed of very few sentences (e.g., page 6, sentence before Two-sample Mendelian randomization analysis).
- Figure 1 could be improved. In its current state, the arrows make it seem like a flow diagram and steps rather than separate analyses.
- Figure 2: please increase the font size of the marker labels.
- Figure 3: please increase the x-axis size so that you do not need to use arrows for the confidence intervals.
- In the discussion, referring to 11% as a modest global genetic similarity is a stretch.
- There is small box on line 375 that should be deleted.
