# Supplementary material for: A comprehensive genome-wide cross-trait analysis of sexual factors and uterine leiomyoma
Source: PLoS Genet. 2024 May 3;20(5):e1011268. doi: 10.1371/journal.pgen.1011268 (PMC11095738; doi:10.1371/journal.pgen.1011268)
Supplement: Attachment — Submitted filename: Response to Reviewers.docx [file pgen.1011268.s014.docx]

**Reviewer #1:** This is a well-written manuscript that explores genome-wide and local genetic correlations between uterine leiomyoma (UL) and two sexual factors: age at first sexual intercourse (AFS) and lifetime number of sexual partners (NSP). The study was carried out using GWAS summary data from European-ancestry cohorts as well as individual-data from the UK Biobank to conduct Mendelian randomization (MR). I have the following questions/comments:

**Response:** We sincerely appreciate the reviewer for their positive feedback on our work and for providing valuable comments that have improved our manuscript. We have carefully addressed each comment and made necessary modifications per the reviewer’s suggestions.

1.	A significant negative genome-wide correlation was found between AFS and UL. In addition, four significant local genetic correlations were identified between these two traits. Do these local correlations have the same direction (i.e., negative) as the genome-wide correlation?

**Response:** We thank the reviewer for bringing this important aspect to our attention. Indeed, all four local regions identified for AFS and UL exhibited negative local genetic correlations. Similarly, the local region identified for NSP and UL exhibited a positive local genetic correlation. These findings are concordant with the results from our global genetic correlation analyses.

To incorporate this, we have provided a comprehensive presentation of the local genetic correlation results for each of the 2,353 tested regions in the **S5 and S6 Tables**, which are shown briefly below.

**…**

**…**

2.	Could the authors speculate a little bit in the discussion about the shared immune and blood-related components between UL with AFS and NSP?

**Response:** We appreciate the valuable suggestion provided by the reviewer. In response to the feedback, we have included a discussion on the biological relevance of immune and blood-related components to UL as well as to AFS and/or NSP. It reads,

“… The similar enrichment patterns when partitioning heritability for UL and that of sexual factors further elucidate potential overlapped etiology related to the immune system. A chronically active inflammatory immune system is suggested to play a role in UL formation (**REF: Yang et al., 2022, PMID: 34741454; Wegienka et al., 2012, PMID: 22608860**), and sexual factors are linked to sex hormones (**REF: Prasad et al., 2014, PMID: 24954690; Najmabadi et al., 2022, PMID: 36186844**) which have well-established impacts on immune responses (**REF: Bouman et al., 2005, PMID: 15817524**). Future work is warranted to fully uncover specific pathways that underlie the UL-AFS/NSP biological associations. …” (lines 369-375 in Revised manuscript (marked-up copy))

3.	Please specify if the pleiotropic AFS/UL SNPs in Table 1 are in the same genomic regions with significant local genetic correlations between AFS and UL. If that is not the case, please speculate why.

**Response:** We appreciate the reviewer for providing the opportunity to make further clarification. In fact, none of the pleiotropic SNPs identified in cross-trait meta-analysis are located in the same genomic regions as the ones showing significant local genetic correlations. We would like to provide further elaboration to explain why this is within expectation and does not undermine the validity of our results.

First, cross-trait meta-analysis aims to identify specific genetic variants, for example, SNPs, that are associated with multiple traits. On the other hand, local genetic correlation analysis quantifies the overall effect of broader genomic regions that encompass multiple genetic variants (i.e., multiple SNPs), rather than focusing on an individual SNP. This **difference in focus and scale of analysis** can result in the lack of overlap between the shared SNPs and the shared regions.

Furthermore, the identification of shared SNPs and regions relies on statistical power, which can be influenced by various factors including sample size, effect sizes of genetic variants, and the average effect size of all variants within a genomic region. It is possible that the cross-trait meta-analysis and the local genetic correlation analysis **have different levels of power to detect significant associations**, leading to the lack of overlap between the identified regions and SNPs. The distinct **statistical approaches** used in each analysis, which were based on non-identical model assumptions, could also contribute to the differences in the detected signals.

Finally, the genetic architecture of sexual factors and UL is highly polygenic, involving a large number of associated genetic components spreading across the genome. Thus, the lack of overlap between the SNP-level signals and the region-level signals might simply reflect the **complex and multifaceted nature of the genetic architecture** underlying the investigated phenotypes.

Taken together, the discrepancy in findings highlights the importance of considering various types of pleiotropy analyses when exploring the shared genetic architectures across complex traits. In our study, the identified global and local genetic correlations, shared heritability enrichments, as well as the pleiotropic SNPs, collectively suggest a shared genetic basis between sexual factors and UL.

We have added a sentence in the **Discussion** section to address this point. It reads,

“… The 10 pleiotropic loci identified for AFS with UL as well as the 5 pleiotropic loci identified for NSP with UL reflected horizontal pleiotropy. **However, it is worth noting that there is a lack of overlap between the identified pleiotropic loci and shared regions, which could be attributed to differences in the focus, scale of analysis, and the power to detect significant associations**. …” (lines 378-382)

**Reviewer #2:** Uterine leiomyomas are a common morbidity in women. They are known to be associated with multiple female reproductive characteristics, including parity and age at menarche. However, the impact of more environment (behavior) reproductive factors, including the characteristics focused on in this paper (age at first sexual intercourse and lifetime number of sexual partners), is unclear. The authors hypotheses that age at first sexual intercourse and the lifetime number of sexual partners could since they could increase individuals’ exposures to infectious agents. They used publicly available genome-wide summary statistics from large European ancestry specific studies of age at first sexual intercourse, lifetime number of sexual partners, and uterine leiomyomas. They performed numerous statistical analyses, including global and local genetic correlation analyses, cell-type-specific enrichment analysis, cross-trait meta-analysis, and one- and two-sample Mendelian randomization. They conclude that their findings provide evidence of considerable overlapping biology, highlighted genetic correlations, clustering of cell-type-specific annotations, and the presence of shared loci across traits. However, they did not find any evidence to support a causal relationship between age at first sexual intercourse and uterine leiomyomas or number of lifetime sexual partners and uterine leiomyomas. The numerous analyses and approach are unique. However, it is not of broad interest to researchers in genetics and genomics and lacks truly rigorous methodology. I finding this manuscript lacking and concerning for multiple reasons which I highlight below.

**Response:** We appreciate the reviewer’s feedback and acknowledge their concerns. We have, to the best of our ability, addressed each of the reviewer’s comment comprehensively and made the necessary modifications as per the reviewer’s suggestions.

Major comments and questions

1.	Though I understand the authors’ logic for taking a genetic approach to this research question (e.g., observational studies are lacking, susceptible to bias and reverse causality, etc.), there are serious concerns about the approach. There are innumerable issues with using genetic instruments to study characteristics (age at first sexual intercourse and lifetime number of sexual partners) that are known to be heavily influenced by the environment. Though the authors discuss the issue of pleiotropy, they do not provide discussion of the enormous effects of the environment (behavior, culture, religion, etc.) on both the sexual factors and uterine leiomyomas.

**Response:** We appreciate the reviewer for emphasizing this important point.

Like the reviewer suggested, while it is true that environmental factors play significant roles in shaping sexual behaviors and UL, the genetic approach offers several advantages in addressing research questions where observational studies may be limited by biases and reverse causality. By leveraging genetic data, our genome-wide cross-phenotype analysis aims to explore the shared genetic basis, pleiotropic effects, and potential causal relationships between sexual factors and UL. It should be noted that this approach allows us to **provide in-depth evidence that could** **complement the existing knowledge on environmental influences**, **rather than dismiss or undermine the role of environmental factors** (**REF: Zhu et al., 2021, PMID: 32693092**).

In fact, **it is common in the literature to utilize genetic information to study the biological underpinnings of factors strongly influenced by the environment, as well as their intrinsic associations with various outcomes**. For instance, a study published in *Nature* (**REF: Okbay et al., 2016, PMID: 27225129**) successfully identified 74 independent genetic loci associated with educational attainment, a complex phenotype influenced by socioeconomic factors, family environment, cultural factors, and educational policies, among others. Another study published in *Nature Communications* (**REF: Sanderson et al., 2019, PMID: 31270314**) leveraged the significantly associated genetic loci to investigate the potential causal effect of educational attainment on smoking behavior, another factor strongly influenced by environmental factors, such as peer influence and cultural norms. Regarding sexual behaviors, a study published in *BMC Medicine* (**REF: Prince et al., 2022, PMID: 35321746**) provided evidence of a significant genetic correlation and causal relationship between **a later age at menarche and a later age at first sexual intercourse**, among other findings. These results highlight the importance of considering a woman’s entire reproductive history from a genetic perspective. It is worth noting that the estimated SNP heritability for educational attainment was approximately 11.5% (**REF: Lee et al., 2019, PMID: 30038396**). In comparison, the SNP heritability estimates for AFS and NSP (the sexual factors that we investigated in this manuscript) range from 12% to 23% (**REF: Mills et al., 2021, PMID: 34211149; Karlsson et al., 2019, PMID: 30643258**).

To further address the reviewer’s concern, **we performed an additional observational analysis** **to examine the phenotypic correlation between sexual factors and UL risk**. Consistent with our primary genetic analyses, we focused on females of European ancestry from the UK Biobank dataset (N=257,414). We defined a diagnosis of UL as the ICD-10 (International Classification of Diseases, 10th revision) codes D25 and the ICD-9 code 218, using linked records of hospital admissions in UK Biobank. To minimize the role of reverse causation, we excluded 16,593 females with a history of UL at baseline or those who were diagnosed with UL within the first two years of follow-up, based on a combination of self-report and clinical documentation. As a result, our final analysis included 240,821 female participants.

We constructed **a Cox proportional hazards regression model**, **taking baseline measured AFS or NSP as the exposure variables**. To minimize confounding and to align with our one-sample MR analysis, we applied two sets of adjustments. Estimates in model 1 adjusted for age, assessment center, the top 10 genetic principal components, educational attainment, and Townsend deprivation index. Model 2 included additional adjustments, building upon Model 1, for age at menarche, age at first birth, ever taken oral contraceptive pill, ever used hormone-replacement therapy (HRT), and age at natural menopause (in the subgroup analysis of post-menopausal females). All analyses were conducted using SAS version 9.4 (SAS Institute, Cary, NC).

We present the results of our observational analysis below for the reviewer’s reference. As expected, **no significant evidence was found to support a phenotypic correlation between sexual factors and the risk of UL**, **corroborating the null findings in our MR analysis that relied on genetic information**.

We acknowledge that genetic instruments do not capture the full complexity of sexual factors and UL, and believe that integrating genetic analyses with robust environmental assessments will provide a more comprehensive understanding of the interplay between genetics and the environment in shaping sexual behaviors and their potential impact on UL risk. We have addressed these points in the **Discussion** section of our revised manuscript. It reads,

“…, although we have carefully selected genetic instruments that show strong statistical associations with the exposures, **common SNPs can only account for a small proportion of the overall variability observed in complex traits**. This limitation is particularly evident in the case of behavioral factors such as AFS and NSP, which are influenced by a multitude of factors, including environmental, demographic, and genetic factors, as well as their intricate interactions (**REF:** **Karlsson et al., 2019, PMID: 30643258**). To obtain a more comprehensive understanding of how genetics and the environment jointly shape sexual behaviors and their potential impact on UL, future research integrating genetic analyses with robust environmental assessments is needed. …” (lines 436-443)

2.	Both the two-sample and one-sample Mendelian randomization demonstrated no causal effects. Yet, the authors heavily highlight the non-causal association analyses. Framing of the results and discussion as common biological mechanisms is misleading as the study found correlation, not causation.

**Response:** We appreciate the reviewer for the opportunity to address their concerns and provide further clarification.

In our manuscript, we stated that our findings do not support a causal association between genetically predicted AFS or NSP with the risk of UL, nor vice versa. Instead, we emphasized that the observed shared heritabilities between sexual factors and UL primarily stem from **overlapping biology**, **rather than** indicating **a** **direct causal relationship**.

It should be noted that our work does not focus on evaluating a “phenotypic correlation” (a separate concept from “genetic correlation”) between sexual factors and UL. The global and local genetic correlations observed simply suggest a shared genetic basis between traits, without necessarily indicating a direct phenotypic correlation as commonly investigated in conventional observational studies.

Applying a similar study design and focusing on genetic associations between migraine and lipoprotein subfractions, the authors of a previous study published in *Neurology* (**REF: Guo et al., 2021, PMID: 34635557**) concluded that, “**Mendelian randomization (MR) estimates were largely null, implying that pleiotropy rather than causality underlies the genetic correlation between migraine and lipoprotein subfractions**.” They extensively explored pleiotropy analyses and discussed the significant genetic correlations and shared loci identified across traits, providing evidence for biological links between triglyceride-rich lipoprotein metabolism and migraine.

As another example, a study published in *Science* (**REF: Brainstorm Consortium, 2018, PMID: 29930110**) examined a wide range of psychiatric disorders and found a substantial level of shared heritabilities among them. The researchers concluded that these findings indicate a strong **interconnection** among common psychiatric diseases and suggest that the current clinical boundaries may not accurately represent distinct underlying disease processes. These findings, importantly, do not imply a causal association between any of the psychiatric traits investigated.

Based on these considerations, we believe our non-causal association analyses offer valuable insights into the shared biological pathways underlying sexual factors and the occurrence of UL, and we have done our best to draft a manuscript where it is unlikely to misinterpret our findings as indicative of a causal relationship.

We appreciate the opportunity to clarify this point and express our gratitude for the reviewer’s feedback.

3.	Ignoring the issues in using genetics for age at first sexual intercourse and number of lifetime sexual partners, I am not convinced that this study would lead to any significant changes in individuals’ decision-making or clinical practice. There is also concern about stigmatization.

**Re: “Ignoring the issues in using genetics for age at first sexual intercourse and number of lifetime sexual partners, I am not convinced that this study would lead to any significant changes in individuals’ decision-making or clinical practice.”** – While we appreciate the reviewer for expressing this concern, we disagree that our study would have no implications for individuals’ decision-making or clinical practice.

Firstly, by revealing shared genetic components at different levels from the whole genome to individual variants, the current study enhances our understanding of a shared biology between sexual factors and UL. With an improved statistical power by combining multiple GWASs, it revealed novel genetic loci jointly associated with both traits, extending findings from previous single-trait GWASs and providing potential therapeutic targets for future drug discovery or repositioning efforts aimed at UL.

Furthermore, considering the extensive research already conducted on the causal effects of unmodifiable reproductive characteristics, such as age at menarche, on the risk of UL, it appears even more important to investigate the potential role of factors that are largely modifiable, including AFS and NSP. Through a comprehensive MR design, the current study for the first time seeks to provide evidence regarding the putative causal relationship between sexual factors and UL risk. The null finding on causation contributes to the knowledge in this important field.

Finally, the current study also demonstrates the value of incorporating genetic analyses and leveraging large-scale datasets to unravel intricate genetic associations. The analytical framework of our research could serve as an example for future investigations into the genetic underpinnings of other complex phenotypes and diseases.

Taken together, we believe that our work provides insights that have meaningful implications for individuals and healthcare professionals involved in the prevention and management of UL.

**Re: “There is also concern about stigmatization.”** – We appreciate the reviewer for highlighting the concern about stigmatization. In our manuscript, we have taken great care to approach our topics with sensitivity and respect. We have been mindful of the language and discussions used to ensure that they do not contribute to stigmatization. Our primary focus has been on understanding the common biological mechanisms and genetic associations between sexual factors and UL, rather than passing judgment or reinforcing stereotypes.

It is important to note that the potential causal effects of sexual behaviors, including AFS and NSP, on a wide range of health outcomes have been extensively investigated in the existing literature. While our study did not find significant associations between AFS, NSP, and UL, previous research has demonstrated adverse health outcomes associated with early age at first intercourse and risk-taking behaviors. These outcomes include sexually transmitted infections (**REF: Hansen et al., 2020, PMID: 31529491, *Acta Obstet Gynecol Scand***), cervical cancer (**REF: Louie et al., 2009, PMID: 19277042, *Br J Cancer***), and even major depressive disorders (**REF: Lu et al., 2023, PMID: 37086791, *J Affect Disord***) and cardiovascular diseases (**REF: Chen et al., 2023, PMID: 37024926, *BMC Med Genomics***).

Considering these findings, we believe that the sensitivity of this topic should not hinder further research aimed at promoting positive health outcomes. It is crucial to continue investigating the potential impacts of sexual behaviors on various health conditions while maintaining a respectful and non-stigmatizing approach. We have addressed this point in our **Discussion** section, where it reads,

“… It is also important to acknowledge the sensitive nature of our work and to emphasize that our intent is to contribute to a larger body of literature aimed at promoting positive health outcomes for women. Stigmatization or judgment of individual sexual behavior has no place in research. …” (lines 463-466)

4.	Also, the SNP heritability of the three highlighted variables, particularly AFS and NSP, is incredibly low. The authors state that the SNP heritabilities (13%, 12-23%, 13%) suggest a non-trivial genetic background for each trait. I would argue that it suggests an even larger role of environmental factors. The second paragraph of the introduction does not provide adequate support for the use of genetics and genetic epidemiologic methods to study these relationships. It undermines it.

**Response:** We appreciate the reviewer’s comment and would like to address their concern.

Firstly, we would like to clarify that the SNP heritabilities mentioned in our study refer to common SNP-based heritability, which represents the proportion of phenotypic variance explained by all variants on a standard GWAS chip (**REF: Yang et al., 2010, PMID: 20562875**).

It is important to note that SNP-based heritability provides an upper bound estimate of the genetic effects that can be detected by a well-powered GWAS. The remaining heritability is likely attributed to rarer and structural variants that have not yet been captured by regular GWAS arrays (**REF: Yang et al., 2015, PMID: 26323059**). For example, the most recent PGC GWAS estimated the SNP heritability of depression to be around 9%, which is only a quarter of the heritability estimated based on twin and family studies (~35%) (**REF: Ormel et al., 2019, PMID: 30877272**). While no twin study has been conducted specifically for sexual factors, **an early twin study reported a heritability estimate of ~30% for UL** (**REF: Luoto et al., 2000, PMID: 11099869**).

The SNP-based heritabilities of 12%-23% (standard errors < 0.003) should not be considered “incredibly low”, especially when compared to those of other complex traits and when found to be statistically significant. For instance, the SNP-based heritability for breast cancer, a complex somatic malignant tumor, was estimated to be approximately 18% (standard error = 0.013) in the largest GWAS conducted on the topic (**REF: Zhang et al., 2020, PMID: 32424353**).

Therefore, while we have acknowledged the significant role of environmental factors in regulating sexual factors and UL (see our previous response), we believe that the SNP-based heritabilities of 3-13%, 12-23%, and 13% should largely support the presence of a non-trivial genetic background underlying each trait. To emphasize the non-negligible heritabilities of our traits of interest to the readers, we have added indications of statistical significance associated with the referenced heritabilities above. It reads,

“… The SNP (single nucleotide polymorphism)-heritabilities of UL, AFS, and NSP have been quantified as 3-13% [CI = 2-22%], 12~23% [CI = 7-28%], and 13% [CI = 12-13%], …” (lines 105-107)

5.	The authors’ argument for the connection between uterine leiomyomas, age at first sexual intercourse, and number of lifetime sexual partners seems to be heavily focused on infection. It is unclear why the authors didn’t instead focus on infection instead of these apparent proxies for exposure to infection. The approach the authors take suggests they are equating earlier age at first sexual intercourse and higher number of lifetime sexual partners to higher risk/more likely to have uterine infections. This is a broad generalization (an individual could have both things but take precautions/use protection and it is heavily dependent on individuals’ partner).

**Re: “The authors’ argument for the connection between uterine leiomyomas, age at first sexual intercourse, and number of lifetime sexual partners seems to be heavily focused on infection.”** – In our study, we presented two possible arguments to support a potential connection between sexual factors and UL. In addition to the hypothesis on intrauterine infection, we also highlighted the possibility that sexual factors and UL may be correlated due to their close link with ovulation and hormonal concentrations (**REF: Prasad et al., 2014, PMID: 24954690; Najmabadi et al., 2022, PMID: 36186844**). This is stated in the **Abstract** section as follows:

“Age at first sexual intercourse (AFS) and lifetime number of sexual partners (NSP) may influence the pathogenesis of uterine leiomyoma (UL) through their associations with **hormonal concentrations** and uterine infections. …”

In the **Introduction** section as follows:

“… In addition to impacting **ovulation and hormonal concentrations** (REF: Prasad et al., 2014, PMID: 24954690; Najmabadi et al., 2022, PMID: 36186844), sexual factors may…”

“…, several common loci have been identified, harboring genes related to **follicle stimulating hormone** (*FSHB*), **implantation** (*ESR1*), and **embryogenesis** (*WNT2*). …”

And in the **Discussion** section as follows:

“… In addition, AFS and NSP are closely linked to **sex hormones** (REF: Prasad et al., 2014, PMID: 24954690; Najmabadi et al., 2022, PMID: 36186844) that have been well-established to impact immune responses (REF: Bouman et al., 2005, PMID: 15817524). …”

**Re: “It is unclear why the authors didn’t instead focus on infection instead of these apparent proxies for exposure to infection.”** – The focus of the current study was to investigate the shared genetic architecture and potential causal links between sexual factors and UL. We apologize for any misunderstanding that may have indicated our intention to investigate the role of infection. To minimize potential misunderstandings and reflect our study’s primary objective more accurately, we have made revisions to the **Introduction** section. The revisions are as follows,

“…In contrast, ~~increased exposure to uterine infections through~~ sexual factors such as age at first sexual intercourse (AFS) and lifetime number of sexual partners (NSP) receive little empirical attention, ...” (line 89)

“…In addition to impacting ovulation and hormonal concentrations, sexual factors may expose participants to infectious agents in the uterus. Therefore, **low AFS or high NSP may correlate with alterations in sex hormones and/or an increased susceptibility to uterine infections**, **both of which** have previously been hypothesized to play an etiologic role in UL pathogenesis. …” (lines 92-96)

**Re: “The approach the authors take suggests they are equating earlier age at first sexual intercourse and higher number of lifetime sexual partners to higher risk/more likely to have uterine infections. This is a broad generalization (an individual could have both things but take precautions/use protection and it is heavily dependent on individuals’ partner).”** – We thank the reviewer for emphasizing this important point. While we completely agree that personal experiences and practices play a significant role in influencing an individual’s risk of infection, numerous epidemiological studies have consistently reported that individuals with lower AFS or higher NSP tend to have an increased incidence of sexually transmitted infections compared to those with higher AFS or lower NSP (**REF:** **Kaestle et al., 2005, PMID: 15800270; Lara et al., 2016, PMID: 26655691)**, even when the scope of sexual experiences was not explicitly limited to vaginal intercourse (**REF:** **Hansen et al., 2019, PMID: 31529491)**. These findings **suggest a broader population-level pattern (rather than an individual-specific pattern as suggested by the reviewer)**, supporting that the relatively crude measures of AFS and NSP may serve as indicators for engaging in risky sexual behaviors that are indeed associated with a higher risk of infections.

While recognizing individual variations, our study aimed to highlight the epidemiological evidence supporting the overall association between lower AFS, higher NSP, and an increased incidence of infections in the uterus. We appreciate the reviewer’s input and acknowledge the need for further research to explore the complex interplay between individual behaviors, protective measures, partners’ information, and the risk of uterine infections in different populations.

6.	The study only included summary statistics from genome-wide association studies conducted in European populations, greatly diminishing the generalizability of the study results.

**Response:** We thank the reviewer for raising this important point.

As we have acknowledged in the **Discussion** section of our original manuscript, one disadvantage of our study is its limited generalizability, as it focuses solely on populations of European ancestry. This limitation arises mainly from the **scarcity of available GWAS summary statistic datasets for other ethnicities**. For example, to the best of our knowledge, **there are** **no GWAS studies specifically on AFS or NSP in non-European ancestry populations**. While we are aware of several GWAS studies on UL conducted in populations of other ancestries, such as the Hellwege et al. (2017) GWAS involving only African Americans, **the corresponding summary statistics have not been made accessible** through the journal or GWAS catalog. We have made diligent efforts to contact the authors of those studies to request data access, but unfortunately, we either have not received responses or the authors were unable to make the summary statistics available.

In addition to the issue of data availability, it is important to note that **the significantly reduced sample sizes in these studies may greatly** **limit our statistical power to detect robust and significant findings** in a genome-wide cross-trait analysis. For instance, the Hellwege et al. GWAS had a total sample size of **8,163** (3,399 UL cases and 4,764 healthy controls), which represents **less than 3%** of the sample size of the Gallagher et al. (2019) GWAS (35,474 UL cases and 267,505 controls). It is also worth mentioning that the developers of *pho*-Hess, a tool for local genetic correlation analysis, recommend **a minimum sample size of 50,000 for reliable results** (**REF: Shi et al., 2017, PMID: 29100087**).

To explore the generalizability of our primary findings, we conducted **an additional observational analysis** examining the phenotypic relationship between sexual factors and UL among female participants of **African ancestry** in the UK Biobank (of note, conducting a one-sample MR analysis among African females is not feasible due to the absence of available African-specific IVs for sexual factors in the existing literature). We employed the same analytical framework as described in our response to comment #1 from reviewer 2. The results of this analysis (shown below) indicated **consistent null findings in the phenotypic association between sexual factors and UL risk**. These findings suggest that the conclusion drawn from this study regarding a null causal relationship is likely applicable to populations of other ancestries.

We agree that conducting further studies using large-scale GWAS that encompass diverse ancestries is crucial to validate the findings of our study. We have revised our manuscript to address this point, where it reads,

“…, we also acknowledge potential limitations. First, as our results were restricted to samples of European ancestry, generalizability to other ancestry groups is precluded. **Studies leveraging large-scale GWAS that encompass different ancestries are required to understand better the genetic associations across diverse populations**. …” (lines 432-435)

7.	The authors utilized publicly available genome-wide summary statistics for NSP that contained females and males, though the outcome they are interested in (uterine leiomyoma) can only occur in those biologically female with uteruses. With no mention of the proportion of the cohort that was male, it is impossible to conclude if their results are biased due to heterogeneity by sex. Furthermore, with the uterine leiomyoma genome-wide association study, there is no discussion of whether females who had hysterectomies were excluded or the percent of the cohort whose phenotype was ascertained by self-report versus clinical documentation (which could introduce bias).

**Re: “The authors utilized publicly available genome-wide summary statistics for NSP that contained females and males, though the outcome they are interested in (uterine leiomyoma) can only occur in those biologically female with uteruses. With no mention of the proportion of the cohort that was male, it is impossible to conclude if their results are biased due to heterogeneity by sex.”** – While we appreciate the reviewer’s consideration on sex-specificity, we believe that using sex-combined GWAS of NSP might only affect our findings to a minimal extent. We would like to present three specific reasons to support our position:

Firstly, the authors of the original NSP GWAS paper (**REF: Karlsson et al., 2019, PMID: 30643258**) stated that there is a genetic correlation of over 80% between the male- and female-specific GWAS for the general risk tolerance trait, which incorporates six supplementary risk tolerance-related phenotypes, including NSP. This piece of result suggests a substantial degree of genetic similarity between sexes regarding the genetic architecture of NSP.

Secondly, we conducted a comprehensive sensitivity analysis using the female-specific summary statistics for NSP obtained from the Neale Lab. This analysis yielded consistent results, indicating both a lack of significant global genetic correlation and a null causal association between NSP and UL.

Lastly, our supplementary one-sample MR analysis was specifically conducted among female participants from the UK Biobank. Findings from this analysis align with those from our sex-combined two-sample MR analysis.

We have explicitly stated these points in the **Discussion** section of our original manuscript as follows:

“…, we conducted our primary analyses using sex-combined GWAS summary data of NSP, which may introduce sex heterogeneity. However, the **high magnitude of genetic correlation** ( = 0.82) between the sex-specific GWASs of the general risk tolerance measure has largely justified the approach of pooling males and females to maximize statistical power; furthermore, **global genetic correlation and two-sample MR analyses using female-specific summary statistics for NSP** (non-formal publication; http://www.nealelab.is/uk-biobank/) yielded largely consistent results ( = 0.03, *P* = 0.50; NSP to UL: OR_IVW_ = 0.88, *P* = 0.50; UL to NSP: OR_IVW_ = 1.01, *P* = 0.50), as in our **one-sample MR analysis restricting to UK Biobank female participants**. Future investigations leveraging sex-specific summary-level data are needed to validate our findings. …” (lines 443-452)

**Re: “Furthermore, with the uterine leiomyoma genome-wide association study, there is no discussion of whether females who had hysterectomies were excluded or the percent of the cohort whose phenotype was ascertained by self-report versus clinical documentation (which could introduce bias).”** – We thank the reviewer for bringing up these important points. For cohorts in which information regarding previous hysterectomy was collected, **the UL GWAS has excluded females who had a history of hysterectomy from all analyses**. We have incorporated this information into the **Methods** section of the revised manuscript. It reads,

“For UL, we obtained association results from… **For cohorts in which information regarding previous hysterectomy was collected, females with a history of hysterectomy were excluded**. …” (lines 150-151)

Regarding UL ascertainment approach, as stated in the UL GWAS paper (**REF:** **Gallagher et al., 2019, PMID: 31649266**), the identification of UL cases was based on **either clinical documentation or self-report**. Detailed descriptions of cohorts and sample selections are available in Supplementary Methods of the original publication. Here, we present the essential information for the reviewer’s reference:

**In WGHS**, “… participants were asked whether they had ever been diagnosed with UL and their age at diagnosis. Cases were defined as women who **self-reported** ‘yes’ to having a history of UL, while controls were classified as women who self-reported ‘no’. …”

**In NFBC**, “… Cases with a history of UL were identified through **national outpatient and inpatient hospital discharge registers** and **self-reported** diagnosis through postal questionnaire at age 46. The hospital discharge registers include WHO ICD codes for identification of disease diagnoses and dates for each hospital visit. Controls were drawn from the rest of the cohort population. …”

**In QIMR**, “… Participants who **answered “yes”** to the “uterine fibroids” option of the question “Have you ever had any of the following conditions?” were selected as cases. … Controls were drawn from twin pairs in the gynecological health study in which both sisters answered “no” to a question about medical history of uterine fibroids (one sample per twin pair). …”

**In UK Biobank**, “… Based on both **hospital-linked medical records and self-report** (interview with research nurse), women with a history of UL were selected as cases, while controls had no previous history of UL. …”

**In 23andMe**, “… Data on participants’ history of UL were collected via **self-report** in online surveys. Medical history of UL was determined with the research question, “Have you ever been diagnosed with uterine fibroids?”, which had three response options: yes, no, and not sure. Females who answered “yes” were selected as cases, those who answered “no” as controls, and those who answered “not sure” were excluded from the study, …”

Therefore, **a proportion of the UL cases included in this GWAS**, particularly those from NFBC and UK Biobank, **were obtained directly from national hospital-linked medical records**. Unfortunately, **without direct access to the individual-level data from all five cohorts**, we are unable to provide information on the exact proportion of cases with clinical confirmation of UL in the GWAS overall. We have **acknowledged the potential for recall bias** resulting from the UL ascertainment approaches as a limitation in the **Discussion** section of our revised manuscript. It reads,

“…To optimize statistical power, our study, as well as the previous UL GWAS (**REF:** **Gallagher et al., 2019, PMID: 31649266**), incorporated UL cases that were ascertained through self-report, introducing the possibility of recall bias in outcome definition. …” (lines 466-468)

8.	The genome-wide association studies for sexual factors both used poorly worded questions to gauge the outcomes, as they did not clarify which type of sexual intercourse they meant or what participants would consider sexual intercourse. The ascertainment methods are particularly crucial for the reviewed study as certain types of sexual contact would likely not increase (or not to the same amount) risk of uterine infections, which the authors appear to argue is the most likely mechanism through which these factors causally impact uterine leiomyomas.

**Response:** We appreciate the reviewer for providing the opportunity for us to further clarify our included datasets. In our study, we leveraged summary statistics from the largest GWAS conducted to date for AFS and NSP. To maximize the statistical power in detecting significantly associated variants, these GWAS utilized data from participants in the UK Biobank. This decision was largely motivated by the **UK Biobank’s significantly larger sample sizes**, which **provides a valuable resource compared to other existing studies that include information on sexual factors**. As far as we are aware, there has been only one GWAS conducted for NSP. While two GWAS have been carried out for AFS (**REF: Mills et al., 2021, PMID: 34211149; Day et al., 2016, PMID: 27089180**), both were performed using data from the UK Biobank.

It is important to note that in the UK Biobank, the specific touchscreen questions used to define AFS and NSP are as follows: “What was your age when you first had sexual intercourse? (Sexual intercourse includes vaginal, oral, or anal intercourse)” and “About how many sexual partners have you had in your lifetime?”, respectively. These questions are outlined in more detail on the UK Biobank website under Category 100056 (<https://biobank.ndph.ox.ac.uk/ukb/label.cgi?id=100056>).

Therefore, the answers provided by the participants may encompass experiences of vaginal, anal, or oral intercourse. We have included this information in the **Method** section of our manuscript, stating:

“AFS and NSP in the UK Biobank were assessed through the questions, ‘What was your age when you first had sexual intercourse? (**sexual intercourse includes vaginal, oral, or anal intercourse**)’ and ‘About how many sexual partners have you had in your lifetime?’ respectively.” (lines 131-134)

As addressed in our prior response, our study primarily **focuses on the broader population-level pattern**, such as a positive correlation between risky sexual behaviors and the risk of infections in the general population. We **recognize the lack of specificity regarding type and context of sexual activity as a limitation** and have revised our **Discussion** section accordingly. It now reads,

“…, we acknowledge that our study’s definition of AFS and NSP, based on the UK Biobank data, includes a broad range of sexual experiences. This lack of specificity regarding the type and context of sexual activity may limit our understanding of the nuanced variations in risk associated with different sexual behaviors. Future research should explore more specifically defined AFS and NSP to assess their impact on the risk of UL and other relevant outcomes. …” (lines 457-463)

9.	There are concerns with the Mendelian randomization analyses. The authors performed bi-directional two-sample Mendelian randomization, evaluating the potential for reverse causation. However, this approach for age at first sexual intercourse is flawed. Age at first sexual intercourse likely occurs early in life (e.g., teens or twenties) for most individuals, whereas, development or diagnosis of uterine fibroid, though the vast majority of females have them by age 50, likely occurs later. The percent of females diagnosed before their first sexual intercourse is undoubtedly very small. This causal analysis is invalid due to this incorrect time/order relationship.

**Response:** We appreciate the reviewer’s thoughtful consideration. We have addressed our motivation for conducting the reverse-direction MR analysis in the **Methods** section of our original manuscript. It reads,

“… Given the prevalence of approximately 4 percent of ultrasound-identified UL occurs in females aged 20 to 30 years (**REF: Evans et al., 2007, PMID: 17555142**), **there is a possibility** **that UL may affect subsequent sexual behaviors**. We therefore conducted a reverse-direction MR to evaluate the putative causal effect of genetic liability to UL on sexual factors ...” (lines 231-234)

We would like to emphasize that the primary goal of our MR study was to examine the potential causal role of sexual factors in the risk of UL. **The reverse-direction MR analysis was primarily adopted as a sensitivity analysis to explore or rule out the possibility of reverse causation**.

10.	In some areas of the paper, the authors use ancestry terminology while other areas they use sociocultural definitions of race and ethnicity (e.g., European ancestry on page 5, white descent on page 8). Their choice in terminology for sex is also flawed, switching between biological sex and gender (e.g., females versus women). Please consider changing the language throughout the paper to the correct terminology.

**Response:** We apologize for any inconsistencies or inaccuracies in our use of ancestry, race, ethnicity, sex, and gender terminology. Throughout the entire revised manuscript, we have now exclusively utilized terminologies related to ancestry (e.g., European) and biological sex (e.g., female).

11.	Mediation analyses would further clarify that the relationships are not being driven by actual biologically controlled factors.

**Response:** We thank the reviewer for suggesting the additional analysis to strengthen our results. Following the reviewer’s suggestion, we performed a comprehensive mediation Mendelian randomization (MR) analysis considering a wide range of potential mediators, including **age at first birth (AFB), age at menarche (AAM), age at natural menopause (ANM), sex hormone-binding globulin (SHBG), sex hormone-binding globulin adjusted for body mass index (SHBGadj), total testosterone (TT), bioavailable testosterone (BT), and estradiol (E2)**.

As presented in the results (shown below), **none of the investigated factors demonstrated significant evidence supporting their role as mediators.** This outcome is expected, given the null total effect of AFS on UL observed in the univariable MR analysis.

Interestingly, we did observe a significant indirect causal effect of AFS on UL risk, **primarily mediated through AFB** (Beta = –0.14, *P* = 0.02, proportion mediated estimated at 120% of the total effect of AFS on UL). While interpreting a proportion mediated above 100% is challenging, this finding may suggest that **any observed correlation between AFS and risk of UL is essentially entirely mediated through AFB**. This **aligns with our meticulously designed multivariable MR analysis**, where a significant causal effect of AFS on UL risk was observed when adjusting for AAM and/or ANM, which, however, attenuated to null when AFB was further included.

We have incorporated the results of the mediation MR analysis in **S9 Table** and have provided a potential interpretation in the **Discussion** section of our revised manuscript. It reads as follows:

“… However, our MVMR exploration results suggest that **this association is predominantly confounded or mediated by the effect of AFB**, **which is further** **supported by the results of a supplementary mediation MR analysis** (S9 Table). …” (lines 408-410)

12.	A follow-up analysis using a study that meticulously defined their cohorts and uterine leiomyomas, such as the genome-wide association studies by Edwards et al or Hellwege et al which contained only cases and controls that had pelvic imaging, would increase the rigor of this study. As is, the study could be biased due to ascertainment of uterine leiomyomas. Since they provide no detail on the proportion whose case status was ascertained via health records versus self-report, the possibility of the study suffering from recall bias cannot be determined.

**Response:** We appreciate the reviewer for pointing this out.

Indeed, utilizing summary statistics from the GWAS studies conducted by Edwards et al. or Hellwege et al. might provide valuable insights into extending our analyses beyond females of European ancestry, as well as evaluating the robustness of our findings in cohorts with stricter definitions of UL. However, as previously mentioned (in our response to **comment #6 from reviewer 2**), despite our greatest efforts, we have no access to the corresponding summary datasets; moreover, these datasets would likely be underpowered for conducting a genome-wide cross-trait analysis.

We have acknowledged the potential for recall bias as a limitation in the **Discussion** section of our manuscript. For more detailed information, please refer to our response to **comment #7 from reviewer 2**.

Minor comments and questions

13.	The **abstract** does not include sufficient information summarizing why the authors hypothesize that age at first sexual intercourse and lifetime number of sexual partners influence uterine leiomyomas. The first sentence of the author summary provides a clear description of the potential mechanisms and should be included in the abstract.

**Response:** We thank the reviewer for this valuable suggestion. We have revised the first sentence in the **Abstract** section. It now reads,

“Age at first sexual intercourse (AFS) and lifetime number of sexual partners (NSP) may influence the growth of uterine leiomyoma (UL) through their associations with hormonal concentrations and uterine infections. Leveraging summary statistics from…”

14.	Important details of the genome-wide association studies that were utilized were not provided or are provided only in supplementary material. More details of the genome-wide association analyses should appear in the manuscript itself, such as what each study adjusted for (principal components, race/ethnicity, age at measurement, etc.).

**Response:** We thank the reviewer for pointing this out. Each GWAS involved in our study controlled for genetic principal components, race/ethnicity, and age at measurement (as reflected by birth year or age at baseline given that information on sexual factors for all participants was collected during the baseline assessment). In the **Methods** section, we have now provided details on covariates adjusted for in each GWAS analysis. The revised text reads as follows:

“… For AFS, we obtained association results from … For NSP, … Both GWAS controlled for the **top ten genetic principal components**, **birth year**, and **sex** (in the sex-combined analysis for NSP). …” (lines 135-136)

“… For UL, we obtained association results from … **Age**, **genetic relatedness**, and **body mass index** were adjusted for in the association analyses. …” (lines 152-153)

15.	More discussion on how the genome-wide association studies defined their phenotype and adjusted their analyses is necessary.

**Response:** We appreciate the reviewer’s comment. In the revised manuscript’s **Methods** section, we have provided detailed descriptions of the phenotypes assessed in each GWAS. The revised text reads as follows:

“… AFS and NSP in the UK Biobank were assessed through the questions, “What was your age when you first had sexual intercourse? (sexual intercourse includes vaginal, oral, or anal intercourse)”, and “About how many sexual partners have you had in your lifetime?”, respectively. …” (lines 131-134)

“… UL was determined based on either self-report (via a single question, such as “Have you ever been diagnosed with uterine fibroids?”) or clinical documentation (e.g., hospital-linked medical records). …” (lines 147-150)

Furthermore, we have included information on the covariates adjusted for in each GWAS analysis, as mentioned in our previous response.

Because our study involves a secondary analysis based on existing GWAS, we have provided only the essential information related to the original single-trait GWAS analyses. For more comprehensive details, we kindly refer readers to the original GWAS papers (**REF: Mills et al., 2021, PMID: 34211149; Karlsson et al., 2019, PMID: 30643258;** **Gallagher et al., 2019, PMID: 31649266**).

16.	In the cross-trait meta-analysis section of the methods, the “Significant pleiotropic SNPs” and “Novel pleiotropic SNPs” do not need to be in bold. The rsIDs in the results also do not need to be bolded.

**Response:** We thank the reviewer for pointing these out. The bold formatting has been removed from the relevant parts of the **Methods** and **Results** sections as suggested.

17.	The authors state they were able to perform separate analyses for pre- and post-menopausal women but did not state how menopause was defined or ascertained. Additional details of these analyses would be useful.

**Response:** We thank the reviewer for bringing this to our attention. The menopausal status of each female participant in the UK Biobank was determined through a touchscreen question, “Have you had your menopause (periods stopped)?”, at the baseline assessment. In our study, participants responded “Yes” were categorized as post-menopausal, while those who answered “No” were classified as pre-menopausal. We have included this information in the **Methods** section, it now reads,

“… Taking advantage of the participant-level data, we were able to perform separate analyses for pre-menopausal (n = 43,741) and post-menopausal females (n = 108,330), categorized based on their response to a single question during the baseline assessment: ‘Have you had your menopause (periods stopped)?’. …” (lines 263-267)

18.	Please consider combining some paragraphs, as some are composed of very few sentences (e.g., page 6, sentence before Two-sample Mendelian randomization analysis).

**Response:** We thank the reviewer for pointing this out. For better flow and coherence, we have revised the manuscript by incorporating the sentence before the Two-sample Mendelian randomization analysis section into the prior paragraph and have improved paragraph structure in the manuscript overall.

19.	Figure 1 could be improved. In its current state, the arrows make it seem like a flow diagram and steps rather than separate analyses.

**Response:** We thank the reviewer for this insightful comment. To present our overall study design more accurately, we have removed all arrows from **Figure 1**.

**Fig 1.** Overall study design of genome-wide cross-trait analysis. GWAS summary statistics for each trait of interest were retrieved from publicly available GWAS(s). A global genetic correlation analysis between sexual factors and uterine leiomyoma was conducted. The estimated global genetic correlation was further dissected at LD-defined regions. Functional annotation analysis within 396 cell-type-specific annotations was conducted on three traits by using genome-wide SNPs. Cross-trait meta-analysis was applied to identify pleiotropic loci, and a comprehensive Mendelian randomization analysis was used to infer putative causal relationships.

20.	Figure 2: please increase the font size of the marker labels.

**Response:** We have increased the font size of the marker labels as the reviewer suggested.

**Fig 2.** Local genetic correlations between uterine leiomyoma and sexual factors. (**A**). Manhattan plot presenting region-specific P-values for local genetic correlation between uterine leiomyoma and age at first sexual intercourse. (**B**). Manhattan plot presenting region-specific *P*-values for local genetic correlation between uterine leiomyoma and lifetime number of sexual partners. Red dots represent loci showing significant local genetic correlation after multiple testing adjustment (*P* < 0.05/2,353). AFS, age at first sexual intercourse; UL, uterine leiomyoma; NSP, lifetime number of sexual partners.

21.	**Figure 3**: please increase the x-axis size so that you do not need to use arrows for the confidence intervals.

**Response:** We have increased the ranges of the x-axis in the forest plots as recommended.

**Fig 3.** Causal relationships underlying uterine leiomyoma, age at first sexual intercourse, and lifetime number of sexual partners. (A). Estimates of causal effects for genetically predicted age at first sexual intercourse with uterine leiomyoma. (B). Estimates of causal effects for genetically predicted lifetime number of sexual partners with uterine leiomyoma. (C). Independent effects of genetically predicted age at first sexual intercourse on uterine leiomyoma after adjusting for each potential confounder separately and together using multi-variable Mendelian randomization. (D). Estimates of causal effects for genetically predicted uterine leiomyoma with sexual factors (age at first sexual intercourse, lifetime number of sexual partners). Boxes represent the point estimates of causal effects, and error bars represent 95% confidence intervals. AFS, age at first sexual intercourse; UL, uterine leiomyoma; NSP, lifetime number of sexual partners; AFB, age at first birth; AAM, age at menarche; ANM, age at natural menopause.

22.	In the discussion, referring to 11% as a modest global genetic similarity is a stretch.

**Response:** We thank the reviewer for pointing this out. Following the reviewer’s suggestion, we have changed the word “modest” to “limited”. It reads,

“… Overall, a **limited** yet statistically significant global genetic similarity was identified between AFS and UL, …” (line 358)

23.	There is small box on line 375 that should be deleted.

**Response:** We have removed the small box from line 375.

**Reviewer #3:** Thank you for this thorough analysis and exploration of sexual factors and UL. I appreciated the complementary approaches taken for this analysis and the summary of methods shown in Figure 1.

**Response:** We thank the reviewer for their positive feedback and for providing instructive comments that could further improve our work. We have responded to each comment, as well as modified our manuscript following the reviewer’s suggestions.

1.	Please provide sample sizes for subgroups of pre- and post-menopausal women.

**Response:** We thank the reviewer for bringing this up. To provide clarity on the sample sizes for the subgroups of pre- and post-menopausal females, we have made the following updates to the **Methods section** of our revised manuscript:

“… Taking advantage of the participant-level data, we were able to perform separate analyses for pre-menopausal (**n = 43,741**) and post-menopausal females (**n = 108,330**). …”

We have also included information on how menopausal status was ascertained, as described in our previous response addressing **comment #17 from reviewer 2**.

2.	It is mentioned that the observed association between AFS and UL may be confounded by AFB. For future research, consider a mediation analysis to analyze the pathway AFS --> AFB --> UL.

**Response:** We thank the reviewer for raising this crucial point and for suggesting additional analysis to strengthen our findings. As per the reviewer’s suggestion, we conducted **a comprehensive mediation MR analysis**, considering a wide range of potential mediators, including AFB. We have thoroughly **incorporated these new findings into the revised manuscript**. For more detailed information, please see our previous response regarding **comment #11 from reviewer 2**.

Typos:
3.	p. 4, lines 105-106; please keep the verbs in the same tense (past or present)

**Response:** We thank the reviewer for pointing this out. Based on the reviewer’s suggestion, the sentence has been updated to:

“…, we **quantified** the shared genetic basis, **detected** pleiotropic effects, and **assessed** putative bidirectional causal relationships underlying AFS, NSP, and UL. …” (lines 118-124)

4.	p. 8, lines 207; “… adjust for potential confounding effect…” should be “to adjust for any potential confounding effects…”

**Response:** The sentence has been updated to:

“…, we further applied an IVW-based multivariable MR (MVMR) approach **to adjust for any potential confounding effects**…” (line 228)

5.	p. 15, line 408; “well-power” should be “well-powered”

**Response:** The sentence has been updated to:

“…due to limited **well-powered** GWAS available for these traits. …” (line 470)

6.	Figure 1: The box “Pleiotropic effect and casual relationship identification” should be “Pleiotropic effect and causal relationship identification”

**Response:** We appreciate the reviewer for bringing this to our attention. We have made the necessary revisions to the wording in **Figure 1** (see our response to **comment #19 from reviewer 2**), as suggested.
