# Supplementary material for: A comprehensive genome-wide cross-trait analysis of sexual factors and uterine leiomyoma
Source: PLoS Genet. 2024 May 3;20(5):e1011268. doi: 10.1371/journal.pgen.1011268 (PMC11095738; doi:10.1371/journal.pgen.1011268)
Supplement: Attachment — Submitted filename: Response to Reviewers.docx [file pgen.1011268.s015.docx]

**Reviewer #1:** I appreciate the authors for addressing all of my comments. The manuscript has greatly improved after these changes.

**Response:** We thank the reviewer for the positive feedback.

**Reviewer #3:** Thank you for your careful attention to the suggestions made. Please include a brief paragraph in the Methods section about the mediation analysis.

**Response:** We thank the reviewer for the positive feedback and for providing additional suggestions. In response, we have included a description of the methods used for the mediation analysis in the main text, with all details provided in the **S1 Text**.

In the main text, we now state (lines 216-218 in the marked-up version of the revised manuscript):

“… Following MVMR, a mediation analysis within a two-step MR framework was performed to explore the potential mediating effects of various hormone-related phenotypes in the sexual factors-UL relationships (**S1 Figure**). Detailed methods can be found in **S1 Text**. …”

In the S1 Text, we have added the following section:

“**Supplementary Methods for mediation analysis:**

To investigate the potential mediating role of hormone-related phenotypes, including age at first birth (AFB), age at menarche (AAM), age at natural menopause (ANM), sex hormone-binding globulin (SHBG), SHBG adjusted for body mass index (SHBGadj), total testosterone (TT), bioavailable testosterone (BT), and estradiol (E2), in the relationship between sexual factors and uterine leiomyoma (UL), we conducted a mediation analysis within a two-step Mendelian randomization (MR) framework (**S1 Figure**).

We obtained the largest available genome-wide association study (GWAS) summary statistics for each mediator under investigation (details on data sources can be found in **S4 Table**). We employed univariable MR to estimate the effect of the exposure trait (AFS/NSP) on the outcome trait (UL risk), referred to as the total effect C, as well as the effect of the exposure trait on each potential mediator, termed the effect A. We applied multivariable MR to assess the effect of each potential mediator on the outcome trait (effect B), while accounting for additional adjustments for the exposure. The proportion mediated (PM), representing the total effect of the exposure on the outcome that is mediated by the mediator, was calculated by dividing the indirect effect (the multiplication of effect A and effect B) by the total effect C. Standard errors for PM were estimated using the delta method. As in our main MR analysis, statistical significance was determined using a Bonferroni-corrected *P*-value threshold of 0.025 to account for the two sexual factors tested.”

Furthermore, we have included a brief summary of the mediation analysis results in the **Results** section (lines 331-335):

“… Mediation analysis did not support any hormone-related phenotypes as mediators in the relationship between AFS/NSP and UL. However, significant indirect effects of AFS on UL were observed through several hormone-related phenotypes, including AFB (**S9 Table**).”

**Reviewer #4:** The authors have conducted a well thought out study and made significant improvements to the manuscript with the revised changes. I have some follow up questions/comments for them to consider.

**Response:** We sincerely appreciate the reviewer for their positive feedback on our work and for providing valuable comments that could further improve our manuscript. We have carefully addressed each comment and made necessary modifications per the reviewer’s suggestions.

1. In the one-sample MR analysis for post-menopausal females, did the authors exclude individuals who had a hysterectomy?

**Response:** We thank the reviewer for pointing this out. We would like to confirm that we have indeed excluded females who have had a hysterectomy from our one-sample MR analysis. To address this point, we have made revisions to the **Methods** section of our manuscript. The revised passage reads as follows (lines 247-255):

“… Taking advantage of the participant-level data, we were able to perform separate analyses for pre-menopausal (n = 43,741) and post-menopausal females (n = 108,330). The categorization was based on a single question asked during the baseline assessment: ‘Have you had your menopause (periods stopped)?’. Participants could choose from five responses, including “**Yes**”, “**No**”, “**Not sure – had a hysterectomy**”, “**Not sure – other reason**”, and “**Prefer not to answer**”. **We classified females who responded “Yes” as post-menopausal, those who responded “No” as pre-menopausal, and considered other responses as missing values, leading to their exclusion from the analysis**. …”

2. The authors state that none of the risk factors investigated in the mediation analysis showed supporting evidence as mediators, but then they also say that they observed a significant indirect effect of AFS on UL through AFB. It is unclear to me how they came to this conclusion when several of the potential mediators they analyzed had indirect effects with a p-value < 0.05. Please clarify.

**Response:** We sincerely appreciate the reviewer for providing us with the opportunity to further clarify our findings.

**Re: “The authors state that none of the risk factors investigated in the mediation analysis showed supporting evidence as mediators…”** – Based on our results, the non-significant findings for the proportion of total effects mediated (*P* > 0.05 across all exposure-mediator-outcome analyses) led us to conclude that none of the mediator variables investigated in our study accounted for a significant proportion of the relationship between sexual factors and UL risk. In other words, we found no evidence to support any of the mediator variables as mediators in the AFS/NSP-UL relationship.

**Re: “…then they also say that they observed a significant indirect effect of AFS on UL through AFB. It is unclear to me how they came to this conclusion when several of the potential mediators they analyzed had indirect effects with a p-value < 0.05…”** – Indeed, although we did not detect significant evidence in support of a mediating role of the potential mediators investigated, we found statistically significant indirect effects of AFS on UL risk through several hormone-related phenotypes (*P*_EffectA*EffectB_ < 0.05/2, number of sexual factors tested), including AFB. The reason why we highlighted AFB in our previous response is two-fold. First, our multivariable MR analysis indicated a potential confounding or mediating role of AFB in the AFS-UL relationship, which was of interest to Reviewer #3. Second, the indirect effect through AFB is particularly strong (Beta = –0.14, *P*_EffectA*EffectB_ = 0.02), compared to the other mediators with significant indirect effects.

We emphasize that two issues need to be taken into consideration while interpreting our mediation results. First, in the AFS-AFB-UL analysis, we observed an indirect effect that is larger than the total effect. This is likely due to **a very small (close to zero) population direct effect of AFS on UL**. In our sample, the null direct effect came out positive due to sample variability, resulting in an indirect effect stronger than the total effect (the sum of indirect and direct effects in opposite directions). This interpretation aligns with our univariable MR analysis, which found no evidence supporting a causal effect of AFS on UL risk.

Second, in analyses involving AFS-AFB/AAM/ANM-UL, we observed significant indirect effects in the absence of a significant total effect. In a mediation model, the standard error of the indirect effect depends on the sample variability of Effect A and Effect B, while the standard error of the total effect depends on the variability of both the indirect effect (including Effect A and Effect B) and the direct effect. With more components involved, there is more uncertainty, making it less likely to find the total effect as significant compared to finding the indirect effect significant. Thus, one possible interpretation for our findings is that **we lack sufficient evidence to demonstrate that AFS influences UL, but if an influence is detected, it is likely due to the intervening effect of AFB** (other factors such as AAM and ANM may also contribute, albeit to a lesser extent), **rather than a direct causal relationship between AFS and UL risk**. This interpretation aligns with our multivariable MR analysis, where the observed significant effect of AFS on UL in the multivariable models became non-significant when AFB was additionally included.

We have included the above two paragraphs in **S1 Text**, under the section titled “**Interpretation and discussion of the mediation analysis results**”. We thank the reviewer for their question and acknowledge that further work is needed to fully disentangle the complex relationships among these sexual factors, reproductive characteristics, and the risk of UL. For a more comprehensive understanding of the relationships between reproductive characteristics (i.e., AFB, AAM, ANM) and UL, we recommend referring to our recent publication that delves into this topic (**REF: Xiao and Wu et al., 2024, PMID: 38191017**).

3. The authors should explain what significance threshold they used for the mediation and MR analyses. They should also consider whether a Bonferroni correction should be applied due to the number of tests performed in their analysis.

**Response:** We appreciate the valuable suggestion provided by the reviewer. Given the primary focus of our MR study is to evaluate the potential causal effects of AFS and NSP on UL risk, we applied a Bonferroni-corrected *P*-value threshold of 0.025 (0.05/2) in all MR analyses, including two-sample MR, MR for mediation, and one-sample MR analyses, to account for the number of comparisons conducted across the two exposure-outcome pairs. The added statement in the **Methods** section reads as follows (lines 257-258):

“A Bonferroni-corrected *P*-value threshold of 0.025 was employed for determining statistical significance in all MR analyses, considering the two sexual factors that were examined.”

4. The methods for the mediation analysis are not described. It would be helpful to understand how the hormone datasets were made since participants in the UK Biobank were recruited between ages 40 to 69. Hormone levels can change with age and the traits being studied can occur at different periods throughout an individual’s life. Do the authors have any thoughts on if this could influence the results?

**Re: “The methods for the mediation analysis are not described…”** – We thank the reviewer for pointing this out. We have added descriptions of the methods and results for our mediation analysis. Please refer to our response to **Reviewer #3**’s comment for the detailed information.

**Re: “…It would be helpful to understand how the hormone datasets were made since participants in the UK Biobank were recruited between ages 40 to 69…”** – In response to the reviewer’s comment, we have provided a comprehensive presentation of the GWAS data source for each mediator investigated in our study in **S4 Table**. In summary, the GWASs of all included sex hormone phenotypes were conducted using data from participants in the UK Biobank, where a wide range of essential biochemistry markers were measured in the blood samples collected during recruitment. Each GWAS has accounted for age at baseline and, if applicable, menopausal status, through adjustments in their regression models. This adjustment helps to isolate the genetic components underlying hormone variation that are largely independent of age differences among the study participants.

**Re: “…Hormone levels can change with age and the traits being studied can occur at different periods throughout an individual’s life. Do the authors have any thoughts on if this could influence the results?”** – We appreciate the reviewer for raising this important point. Although our study does not primarily focus on sex hormones, we acknowledge the potential influence of age-related hormonal changes on our mediation analysis results, particularly for AFS.

Ideally, measuring sex hormones during the period when individuals typically experience their first sexual intercourse would provide a more direct assessment of the potential mediating effect. However, in the case of UK Biobank participants, the median AFS is 18, while the median age at recruitment (when sex hormone levels were measured) is 57. We acknowledge this limitation and recognize that our inability to perform participant-level analysis using sex hormone levels measured among younger populations, or summary-level analysis using age-stratified GWAS data for sex hormone traits, is due to data constraints.

It is important to emphasize that future studies leveraging data from participants within the specific age group in question are necessary to refine our understanding of how sexual factors, sex hormones, and UL risk interact during different life periods.

We have addressed this limitation in **S1 Text**, under the section titled “**Interpretation and discussion of the mediation analysis results**”. In the main text, we have included the following sentence (lines 398-400):

“…, which is further supported by the results of a supplementary mediation analysis (**an interpretation and discussion of the mediation results can be found in S1 Text**). …”

5. UK Biobank data was used for both the UL and sexual factors GWAS. Having overlapping samples in the two-sample MR analysis when non-overlapping samples are required, could greatly bias the results. Although this was briefly discussed as a limitation, considering the impact of this, it should be thoroughly addressed. Did the authors explore any ways to avoid this, such as by using a leave-one-out analysis for the UL GWAS or using an independent dataset?

**Response:** We thank the reviewer for this comment.

During our analytical stage, we attempted to access GWAS datasets that did not involve samples from the UK Biobank to mitigate the issue of sample overlap. However, to the best of our knowledge, only one GWAS has been conducted for NSP, and it utilized data from the UK Biobank. While two GWAS have been performed for AFS (**REF: Mills et al., 2021, PMID: 34211149; Day et al., 2016, PMID: 27089180**), both studies utilized data from the UK Biobank. Similarly, although several GWAS on UL have been conducted among European populations, a large proportion of their participants were from the UK Biobank, and results without UK Biobank data are not publicly accessible.

While it is currently infeasible to perform a validation analysis using independent datasets, we maintain that the issue of sample overlap would not significantly impact the conclusions drawn from our study. For analyses investigating the potential role of pleiotropy, **the employed approaches were specifically designed to mitigate bias introduced by sample overlap**. In terms of causal inference analyses, any bias resulting from sample overlap in two-sample MR could potentially lead to a false significant association, which was not observed in our analysis. Additionally, our one-sample MR analysis conducted exclusively within the UK Biobank cohort yielded similar null causal effects, further **addressing concerns regarding sample overlap in two-sample MR**.

We have revised the **Discussion** section of our manuscript to highlight the need for future validations. It reads (lines 442-446),

“…, a considerable proportion of sample overlap may exist among the GWAS datasets used in the current study. However, the genetic approaches (i.e., LDSC, SUPERGNOVA, CPASSOC) we applied are not subject to bias introduced by sample overlap, and any bias caused by sample overlap in two-sample MR may falsely lead to a significant association, which was not observed in our analysis. **Future validation using independent datasets is warranted**.”
